# Meaningful public involvement: changing research institutions toward epistemic justice

**DOI:** 10.3389/fpubh.2025.1655189

**Published:** 2025-09-19

**Authors:** Tomasz Krawczyk, Jan Piasecki, Jacqueline Galica, Marcin Waligora

**Affiliations:** ^1^Department of Bioethics and Health Psychology, Faculty of Health Sciences, Jagiellonian University Medical College, Krakow, Poland; ^2^Research Ethics in Medicine Study Group (REMEDY), Jagiellonian University Medical College, Krakow, Poland; ^3^Doctoral School of Medical and Health Sciences, Jagiellonian University Medical College, Krakow, Poland; ^4^Division of Cancer Care and Epidemiology, Queen’s Cancer Research Institute, Kingston, ON, Canada; ^5^School of Nursing, Queen’s University, Kingston, ON, Canada

**Keywords:** public and patient engagement, public and patient involvement, institutional change, epistemic justice, knowledge space, research institution, research ethics

## Abstract

Public and patient involvement and engagement (PPIE) in research is increasingly expected and often formally required by the sponsors. However, creating and sustaining conditions for meaningful PPIE can be challenging. It requires efforts of all involved parties. While much attention is given for the ethical inclusion of individuals as research participants, their collaboration with researchers and design of accessible research processes, there is a question of how research institutions can support PPIE. We argue for comprehensive changes within research institutions to facilitate meaningful PPIE practice. These changes should include institutional culture and attitudes toward the public members involved in research, to foster meaningful encounters between people with different forms of knowledge and life experience, such as professionally trained researchers and members of marginalized social groups. In this study, we propose a framework of institutional changes for PPIE, which focuses on their sociocultural and epistemic features. We explore the context of PPIE and possible risks related to disregarding public members as owners of valid knowledge. We use the *order of change* model as a frame and emphasize the role of *third-order changes*, which involve raising awareness about diverse forms of knowledge. Such changes would allow for sustaining PPIE research as *knowledge space,* wherein public members and researchers can respectfully share knowledge to inform scientific inquiries. Based on these conceptualizations, we outline practical examples and future directions. Better conceptualizing of institutional changes can contribute to facilitating their implementation and thereby more ethical research practice.

## 1 Introduction

In recent decades, the importance of involving lay people in research has become widely acknowledged within scientific communities. A variety of terms, coming from different research traditions and world regions, have been used to describe such practices (1–3). In this study, we use the term *PPIE*—*public and patient involvement and engagement*—as an umbrella term for meaningful research practices “carried out ‘with’ or ‘by’ members of the public rather than ‘to’, ‘about’, or ‘for’ them” (4). We also use the term *public* to collectively describe patients, their relatives and friends, research participants, and others affected by research and healthcare. As expectations for PPIE in contemporary research continue to rise, there is a growing attention to the sociocultural context of knowledge creation (5, 6). However, the primary focus is on the individuals involved and the research process (7–9). Fewer authors discuss structural and institutional issues, such as the impact of dominant scientific paradigms, institutional culture, or collective biases in academia (10–12). Thus, there is a question of how research institutions should adapt to foster PPIE.

Our main claim is that not only involved public members and individual researchers but also research institutions should change themselves to facilitate meaningful PPIE. We argue that deep institutional changes are necessary to create and sustain *knowledge spaces*. We define *knowledge spaces* in accordance with sociology of medicine as contexts wherein different forms of knowledge interact in a dialog on a possibly equal basis with the aim of enhancing understanding and foster scientific progress (13), p. 1113; (14), p. 544. The different forms of knowledge may consist of experiential knowledge, possessed by people with lived experience of the condition or circumstance under study, and professional knowledge owned by clinicians or scientists (15, 16). Knowledge spaces require the research project to become genuine collaboration between researchers and public members wherein both sides exchange their perspectives and examine existing biases. They would allow for more ethical and reliable PPIE research, contributing to, among others, broadening knowledge with the first-person experiences and empowering those in epistemically subaltern positions (17). For example, in psychology research, a team of psychotherapists can establish a participatory project with autistic people which focuses on the design of psychotherapeutic intervention for neurodivergent patients. In such project, the perspective of neurodivergent people and their experiences can be included and discussed toward increasing mutual understanding and supporting implementation of the intervention. Creating such spaces may require changes in *epistemic resources* of involved parties. *Epistemic resources* are the interpretive frameworks or cognitive schemata consisting of language, concepts, and meanings, as well as judgment rules (18), pp. 717–718. These resources guide the way how individuals approach the world and enable them to make sense of their experiences. For example, Deaf people who identify as members of Deaf culture can differently perceive both deafness and hearing and rely on spatial–visual mode of cognition; thereby, they can develop different epistemic resources than hearing majority (19). Such knowledge space would enable both Deaf people and researchers to express their knowledge, experiences, and expectations about the issue in question in an open, respectful manner. We argue that to enable and sustain such practices, comprehensive changes are necessary—changes which would also affect the epistemic resources of research institutions.

The aim of this conceptual article is to explore the epistemic and sociocultural basis for institutional change in support of meaningful PPIE, which we pursue through four specific objectives:

- To analyze the epistemic context of PPIE, including risks such as *epistemic injustice* (20, 21)—wrongs related to knowledge inequalities and disregarding public members as owners of valid knowledge;
- To define the conditions for knowledge spaces using the institutional *order of change* model (20, 22);
- To conceptualize research institutions as collective agents capable of transformation—formal social entities with a sense of group agency;
- To examine current initiatives and outline possible future theoretical and practical directions.

Through this exploration, we aim to provide a conceptual framework for analyzing changes in research institutions. We ground our analysis within the feminist, sociology of medicine, and critical disability literature, focusing our interest on the sociocultural context of creation and exploitation of knowledge. We situate our framework in health sciences research but acknowledge that it can be relevant also to other fields.

### 1.1 Positionality statement

We are researchers (psychologist, registered nurse, bioethicists) from Poland and Canada. We recognize our epistemic limitations in regard to underrepresented groups’ perspectives, as well as privileged positions in regard to social status and access to education and knowledge. Jointly, we have experience in research ethics, social science research, and PPIE research. Our reflection on PPIE is influenced by decolonial thought and the notion of epistemic diversity, which seeks to uncover, recognize, and position other—non-mainstream—forms of knowledge (12), p. 2513. We assume that all forms of knowledge are partial and situated within specific temporal and spatial contexts, whether it is the professional scientific knowledge or that one owned by the patients and members from marginalized communities. Our point of view is open to examination by the perspectives of members of marginalized groups, as well as researchers from other fields.

## 2 Sociocultural and epistemic context of PPIE

One of the broad approaches defines PPIE^1^ as “the active, meaningful, and collaborative interaction between patients and researchers across all stages of the research process, where research decision making is guided by patients’ contributions as partners, recognizing their specific experiences, values, and expertise” (1), p. 682.

**Figure 1:**
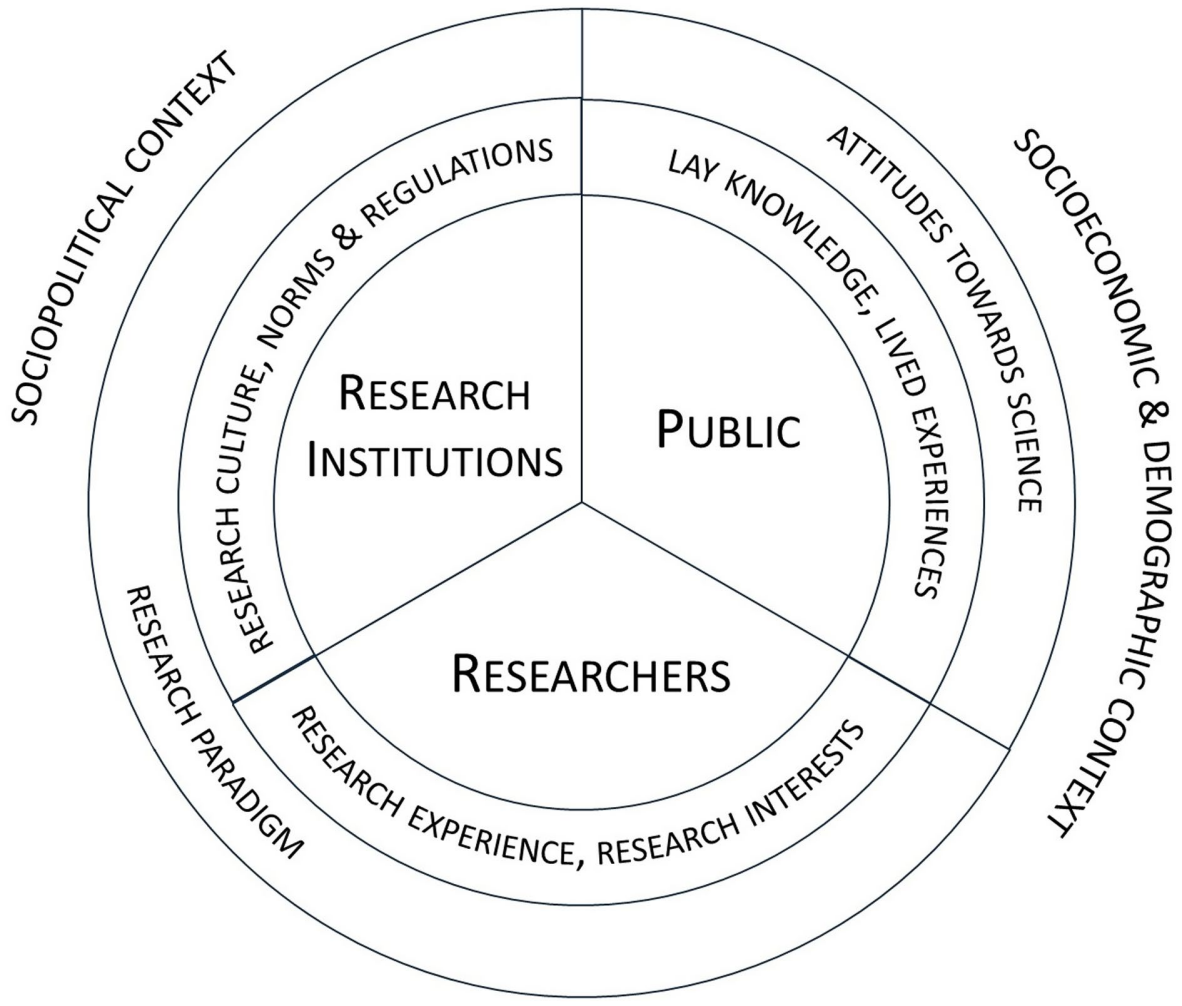
Overview of the agents involved in the PPIE process and the factors influencing their performance.

Concerning the involved agents, interactions within PPIE entail encounters between two groups of people with different backgrounds: *patients,* or more broadly the members of *public;* and *researchers*^2^. These encounters happen in specific context—during the research process which takes place in specific research setting. Vincent et al. (23) p. 1–2 situate the relationship between researchers and members of public—*working relationships*—in the context of the *culture of engagement in research institutions*, which is shaped by the *dominant (health) research paradigm context*. While research decision-making in PPIE should be “guided by patients’ contributions” (1), p. 682, the rules of research processes are—by default—defined and organized by scientific rigor, researchers, and the organizational structure of research institutions (11, 23). Drawing upon the literature on collective agency (24), we propose to distinguish three kinds of agents involved in PPIE research: *(i) public, (ii) researchers,* and *(iii) research institutions—*collective agents which govern the research process (Figure 1). We understand *research institutions* as complex social entities, operating under rational group-level decision-making procedures (24)—universities, medical colleges, or other organizations which conduct research. The public, researchers, and research institutions may share different epistemic resources, which, are the interpretive frameworks, cognitive schemata (concepts and meanings), as well as judgment rules and cognitive procedures (18).

The public and researchers may possess not only different epistemic resources but also different values, life experiences, priorities, goals and interests, as well as have various levels of education, conceptual fluency and attitudes toward science (25). Furthermore, research institutions may vary depending their institutional, regulatory, and political contexts. They may follow different scientific paradigms, which are related to specific values, goals, and concepts (26, 27). In PPIE processes, these various backgrounds intersect which may have multiple effects. On the one hand, PPIE may have a beneficial impact on research, being a means to achieve various objectives. Harting et al. (17), pp. 11–15 reviewed the available rationale for PPIE, which they grouped into five categories. First and second, respectively, are *democratic rationale* and *consumerist rationale,* which concern the normative and economic rights of citizens to “have a voice” in research. These rationales highlight the intrinsic value of PPIE and economic interests of citizens—taxpayers. Third, a *transformative rationale* emphasizes the efforts to “empower marginalized groups” and express their voices through research. The last two, *substantive rationale* and *instrumental rationale*, focus on improving research quality, gathering rich contextual knowledge, as well as increasing research efficiency and applicability. PPIE and partnership approaches are also postulated as a means to achieve epistemically just healthcare and research (28).

On the other hand, PPIE takes place within hierarchical structures that may suppress perspectives that contradict mainstream scientific paradigms (6, 11). Thus, PPIE is also prone to various harms and wrongs to involved public members as epistemic agents—active contributors to and users of knowledge. For example, researchers may distrust experiential lay knowledge, question the status of public members as knowledge owners (29) or lack awareness, willingness, and resources to engage properly (9, 30). In addition, institutions which guide the research process can be opaque (31), constituting complex and demanding settings. This may lead to marginal or tokenistic engagement—symbolic or superficial processes, intended to satisfy the imposed requirements rather than foster genuine collaboration (32). Moreover, the aforementioned PPIE rationales can conflict with each other. They may differ in power and recognition regarding the context (33). For example, during joint discussions, the influence of participating institutional funders or professionals may outweigh the voice of non-profit organizations that support patient rights, which in turn may overshadow the perspectives of individual patients (34). Furthermore, different agents who argue for PPIE can share distinct (sets of) rationales. For example, research authorities may consider primarily *substantive* and *instrumental* reasons. They may emphasize research applicability and prioritize gathering extensive data on how the intervention functions across various settings. The public, however, may focus primarily on *democratic* or *transformative* rationales, placing greater value on opportunities to be heard by professionals and express their experiences of recovery. Front-line researchers may share all these rationales, stepping into a dual—researcher-activist—role (35). However, this discussion exceeds the scope of our study.

Despite its empowering character, PPIE can inadvertently result in establishing unequal and harmful relations within collaborative research initiatives (33, 34). These relations may be mutually maintained if some benefits, such as access to better healthcare, may be acquired by the public members involved in research processes (11). In addition, existing institutional structures can perpetuate power hierarchies, hindering respectful knowledge exchange and mutual learning (31, 36). Such dynamics may easily place involved people in unjustly subordinate positions toward researchers, regardless of whether they belong to marginalized groups. Unjust treatment may also arise in terms of perceived credibility of public members and their capacities to produce, process, and exploit knowledge. In what follows, we outline these risks within PPIE through the conceptual lens of *epistemic injustice*.

### 2.1 Epistemic injustice in PPIE

The concept of epistemic injustice originated from the Fricker’s work (37). Since then, it has been discussed within a range of contexts, among them healthcare and research (6, 31, 38). Epistemic injustice is defined as the wrong related to knowledge and people’s capacities as independent and active knowledge users—*epistemic agents* (37), p. 1. It can occur in several ways, to specific individuals, by disrupting cognitive processes or through systematic perpetuation of power differences (39). In the context of PPIE, two forms of epistemic injustice are particularly relevant: *participatory epistemic injustice* and *epistemic trust injustice* (6), pp. 315–319; see also: (40), which reflect two aspects of knowledge sharing. The first relates to whether an individual is perceived as capable and worthy of participation in knowledge generation. For example, a woman with Down syndrome from a small town may not be asked to participate in research because of a preconception that research is “too demanding” for her. The second aspect of knowledge sharing concerns whether an individual has the necessary conditions to receive knowledge and whether she perceives scientists as trustworthy. For example, an older Roma man may perceive researchers as representatives of dominant cultural group that once forbade him to use his mother tongue at school and subjected him to stigma. Consequently, he may decline participation in research out of a broader mistrust toward the dominant group. Epistemic injustice can occur at various levels. We will analyze them following the Dotson’s account (20, 21). Dotson (20, 21), (Table 1) distinguishes three kinds of epistemic injustice: *testimonial, hermeneutical,* and *contributory injustice*, structuring them within a heuristic of the institutional *order of change* model (22). This account allows for conceptualization of unjust epistemic practices within institutions in a gradual manner, regarding the scope of change required to oppose them. We find this approach promising for discussing the epistemic impact of research institutions on PPIE processes.

**Table 1:** Overview of the forms of epistemic injustice with examples from the PPIE practice.

Injustice	Definition	Examples from PPIE
Testimonial injustice	Injustice related to the credibility given to an individual’s words, based on a prejudice (37)	Disregarding patients’ first-person experiences and testimonies (10, 29): A patient-coresearcher in clinical trial is not believed that he felt pain, because he was given proven painkiller medication, prescribed by experienced practitioner.
Hermeneutical injustice	Injustice related to a gap in epistemic resources which prevents an individual from effectively naming and expressing her experiences. Both speaker and receiver lack resources to name the experience (37)	Gaps in scientific knowledge concerning subjective experiences of illnesses or disabilities (29, 82): The type of pain experienced by an autistic woman does not align with the symptom descriptions in medical charts, making it difficult for her to express her experiences.
Contributory injustice	Systematic depreciating of individual’s epistemic contributions due to structural biases of dominant epistemic resources and ignorance of those who share them (21) Unlike hermeneutical injustice, there are multiple sets of epistemic resources with different interpretive content. Speakers are able to name their experiences but these are not recognized within the dominant set of epistemic resources	Lack of recognition of minority groups’ epistemic resources group (42): Deaf people are perceived by the hearing majority as a disability group. However, many Deaf people consider themselves as a socio-linguistic minority, oppose the disability categorization and develop their own visual–spatial Deaf epistemology. Their claims and experiences may be unrecognized by healthcare practitioners. This may result from differences in used epistemic resources: concepts (e.g., Deafhood vs. hearing loss) and meanings (e.g., embracing Deaf culture vs. need for speech therapy), and the ignorance to see and understand the other perspective.

In *testimonial injustice,* there is a prejudice related to the identity of an individual (speaker) which negatively influences her perception as a knower (37). Research and healthcare settings add extra layers to such prejudices. Public members may be judged not only as members of specific social group, e.g., “women or gays”, “lay people” who do not possess professional knowledge (6). Thus, their testimonies—contributions to shared knowledge—may be easily disregarded when not supported by other existing evidence. Disregarding lived testimonies in this way may lead participants to self-silencing, which can further diminish their active engagement in research processes (40).

*Hermeneutical injustice*, in turn, involves more complex situations where collective epistemic resources are deficient themselves—even with gaps concerning apt resources or having some resources distorted. This leaves some groups with a “significant area of [their] social experience obscured from collective understanding owing to a structural identity prejudice in the collective hermeneutical resource” (37), p. 155. PPIE is prone to such injustice. For example, evidence-based approaches have inadvertently led to the preference of generalizable data from large studies over contextualized patient’s (and also clinician’s) knowledge (41). It constrains the spectrum of “apt” terminologies in the field. Thus, patients may struggle to express themselves and meaningfully contribute to the research.

Finally, *contributory injustice* broadens the understanding of epistemic resources—that is there is no single, universal set of epistemic resources (21), p. 31. Each social group can possess its own set of epistemic resources, which may differ in recognition and status within social power relations. Furthermore, individuals can be (willfully) ignorant in utilizing their own set of epistemic resources, disregarding those of others (18). This perspective stresses the collective experiences of epistemic injustice in a virtue of belonging to a marginalized group (42). Such groups share similar experiences, which can articulate and interpret them, but those interpretations fall outside the mainstream set of epistemic resources, as in the example of autistic or Deaf people (see Table 1).

One of the rationales for PPIE concerns the empowerment of underrepresented groups to express their voices (17) and therefore counters epistemic injustice. However, PPIE is simultaneously prone to such injustice. The good will, commitment, and openness of public members and frontline researchers are of utmost importance in PPIE but may be insufficient to mitigate epistemic injustice and sustain favorable conditions for knowledge spaces. There is a need for structural remedies to efficiently counter the structural character of many forms of epistemic injustice, as well as their cumulative effects (43, 44). Addressing epistemic injustice at various structural and institutional levels may require differentiated approaches and tailored strategies. Here, we will use the *order of change* model (20–22) as a frame to outline three scopes of change within research institutions that can facilitate the establishment of knowledge spaces and related forms of epistemic justice.

## 3 The *order of change* model

The *order of change* model is a framework designed to portray how meaning assigned to certain experiences can be altered within organizational social structures (22). The term *order* reflects the idea that meaning can be changed in a gradual manner, differing in regard to the scope and depth of the change. The model distinguishes three ways—*orders—*of implementing changes. Dotson (21) presented these orders of change as corresponding to increasingly complex forms of changes required to counter presented three forms of epistemic injustice (see Table 2). The model uses a cognitive sciences’ concept of *schemata*—mental “templates, that when pressed against experience, give it form and meaning” (22), p. 484. These can be created, shared, and negotiated also within social groups, such as members of organizations, to guide collaborative actions and interpretations of the events. As such, *organizational schemata* are a kind of “shared epistemic resource like language [concepts and meanings] that enables goals and pursuits to be shared collectively” (20), p. 118. *Schemata* are generally stable and resilient to change (18); however, they can be altered when they prove insufficient for guiding how an organization engage the world. Consistent with Dotson’s approach (20, 21), we broaden the analysis from the level of *organizational schemata* to that of *shared epistemic resources*.

**Table 2:** Overview of the *order of change* model (22) with examples from PPIE practice and related forms of epistemic injustice.

Change	Definition	Related epistemic justice	PPIE examples
First-order	Altering behavioral strategies to better fulfil current needs, according to the existing epistemic resources	Testimonial justice Redistributing the credibility among social groups, aiming to ensure its fair share, nurturing reflexive testimonial sensibility (21).	Training the research team to pay more attention to patient opinions during the study, e.g., a clinical trial. Developing a broad recruitment strategy for a clinical trial to reach diverse populations.
Second-order	Altering institutional epistemic resources to enhance understanding and effectively address current needs	Hermeneutic justice Developing new concepts and meanings, altering existing conceptual frameworks to reduce gaps in epistemic resources (37).	Recruiting a number of patient co-researchers to broaden the perspective on the topic in question. Conducting a participatory study aiming to discuss understanding of research’s meaning and used terminology (83).
Third-order	Raising awareness about various sets of existing epistemic resources, learning how to transcend one’s owned epistemic resources and be able to shift among various their sets depending on the needs	Contributory justice Mitigating disparities between distinct sets of epistemic resources, ensuring equal status allocation between them, practicing epistemic apprenticeship (36). Empowering marginalized social groups, ensuring their participation in creation of knowledge (21).	Transformation of a healthcare research institution which conduct research on deafness; recruitment of both Deaf and hearing research and administration staff, reflecting newest medical and Deaf studies knowledge, involving staff at each level into the (research) accessibility activities, employing the *truth and reconciliation model* into research projects conducted with Deaf community members (52).

*First-order* changes are (relatively) easy to introduce into the institution since they do not require going beyond existing epistemic resources. In the context of epistemic injustice, a change toward testimonial justice also does not need to question the concept of credibility given to a specific social group (21). First-order changes for PPIE may involve revising existing institutional guidelines on research conduct to enhance active participation of the public. For example, research institutions may instruct researchers to conduct research in community spaces or hire interpreters for Deaf or Latine participants (45, 46). Similarly, researchers may provide plain language summaries of publications translated into minority languages (47). First-order changes may also include modifying existing research methods, for example, adding qualitative studies to large quantitative projects and extending the spectrum of research techniques (27). Such processes do not require to directly affect institutional epistemic resources and may have little impact on how the whole institution functions. However, they can be formalized as guidelines or educational resources (48–50) and be a starting point for introducing *second-order* changes.

*Second-order* changes address insufficiencies of existing epistemic resources—hermeneutic injustice—by filling conceptual gaps, thereby enabling all individuals to express themselves more effectively (21). New terms and meanings can be incorporated into vocabularies used in research to facilitate and broaden mutual understanding between public members and researchers. This requires reshaping research culture. PPIE itself can be described as a second-order change. The public members are invited to express their perspective, name their experience which broadens knowledge about the researched issue [see *substantive rationale;* (17)]. It is embodied in, for example, collaborative design of research projects, data gathering, or interpretation of the results (45). Second-order changes may also increase efforts to reach participants before the study to establish relations and mitigate mistrust—an issue of utmost importance when working with marginalized groups (51, 52). Moreover, institutions may act to increase overall ethical awareness of researchers (53, 54). Various approaches are developed to facilitate the exchange of thoughts among research teams (55–57). Such processes transform epistemic resources of research institutions and alter their functioning, especially on the level of research teams. However, these changes may not be strong enough to influence the institutional structures. Researchers who introduce PPIE projects may face procedural barriers and struggle emotionally because of the institutional resistance (30). When no senior staff or authorities support the changes, there is also a risk that such PPIE projects would be perceived as partial or insincere, especially in projects with groups which have been historically oppressed in research (11, 52). Hence, there is a need for *third-order* changes in research institutions.

*Third-order* changes enable agents to recognize the plurality of schemata and move beyond their particular sets. They effect in a meta-awareness of being situated within a specific set of epistemic resources, one among the others. Thus, third-order changes result in a competence of moving between various (previously recognized) sets of epistemic resources, according to the institutional needs. Such competence can help counter contributory injustice by addressing disparities between distinct sets of epistemic resources and the unequal attribution of status among them (21). Third-order changes may provide space for those who share epistemic resources other than the mainstream ones. We argue that such changes are necessary, but also not sufficient, to ensure sustainable implementation of PPIE at research institutions and establishing them as knowledge spaces. Now, we will outline how research institutions can be conceived as collective epistemic agents—social entities with a sense of group agency, who can both obstruct and facilitate reaching epistemic justice in PPIE.

## 4 Research institutions as collective agents

The way social groups and organizations are formalized influences how we understand their features and the responsibilities we attribute to them (24). It also shapes the strategies needed to alter their shared epistemic resources, including the strategies required to counter epistemic injustice. Institutions are formalized, *highly resilient* social structures, “composed of cultural-cognitive, normative, and regulative elements that […] provide stability and meaning to social life” (58), p. 6. In addition, research and academic institutions have distinct characteristics, being “loosely coupled systems with diffused decision making, as well as goal ambiguity” (59), p. 120; (60). These features facilitate introduction of minor changes but make institutions to struggle when major, transformative changes are needed. We argue that such major changes are needed to reorientate a research institution in a new direction, such as embracing the PPIE approach.

Any major institutional change should be structurally holistic, combining both bottom-up and top-down movements (61), and impact institutional structures, processes, and actions (59). Complex activities are needed to affect research personnel at all institutional levels, as well as institutional procedures, goals, and values, ensuring that the change is sustainable (59, 61). Such changes require various resources, including financial, workforce, and time. We argue that, for changes seeking to embrace PPIE approach, among these factors the necessary—but not sufficient—condition is a change within institutional epistemic resources: raising awareness of and recognizing diverse forms of knowledge owned by various non-dominant social groups (i.e., neurodivergent people, people with chronic illnesses or ethnic minorities). The change should also interact with and be grounded in the *institutional ethos* (60). Fricker (61), p. 91 conceptualizes the *institutional ethos* as “collective motivational dispositions and evaluative attitudes within the institutional body, of which the various good or bad ends orientate the institution’s activities.” Similarly to the character of an individual, these dispositions and attitudes occur regularly and are stable within time. For example, an institution can articulate the will to respect diverse forms of knowledge in the institutional mission and formalize within decision-making procedures which motivate actions of its members. Institutions can thereby influence and shape behaviors of their members, to ensure alignment with institutional goals—a dynamic that is crucial for their proper functioning.

We may ascribe certain features to institutions—collective agents, as their structure and procedures promote some and discourage other behaviors among their members (62). We draw on a *non-summative account* of collective agency, according to which institutions, as formalized groups, are not reducible to the sum of their members, thus can be considered as bearers of collective agency (62), pp. 2–4. For example, an institution can exhibit a feature even if most of its members do not possess that feature individually; a research institution can remain opaque for participants, even when all its members act in a most direct manner (31). Institutional features may include shared values which constitute institutional ethos, including epistemic virtues and epistemic vices. Epistemic virtues aim at achieving epistemic goods, such as truth or understanding, for example, *curiosity* and *humility*. On the contrary, epistemic vices—“culpable failures of epistemic virtue,” like *hubris*, *insouciance* or *arrogance*, hinder acquisitions of the epistemic goods (61), p. 98. In PPIE, epistemic virtues and vices lay at the intersection of the pursuit of truth—gathering more accurate knowledge—and the pursuit of good—respecting public members as knowledge owners. These two aspects are deeply interconnected, as active participation of minority members may contribute to both broadening mutual knowledge and mitigating future harms, thus contributing to epistemic justice (17, 36).

Epistemic vices of research institutions can contribute to research misconduct and questionable research practices, rendering such errors institutionally grounded (63, 64). Moreover, vicious institutional ethos can conduce moral transgressions with procedural or structural factors, which can increase motivation of individual members to commit various kinds of moral transgressions (63). For example, the failure to acknowledge the neurodiversity perspective within a medical faculty may contribute to the dismissal of autistic people’s research experiences that diverge from the mainstream medical view. Moreover, it may lead to further marginalization of autistic people in healthcare by professionals who graduated in that faculty. This can limit both scientific knowledge by excluding autistic people’s perspectives and their trust in healthcare research, thereby causing social harms. Therefore, structural solutions should be implemented to foster and sustain virtuous practice at both institutional and individual levels (42, 44). Specifically, respectful PPIE research is unlikely to occur unless research institutions establish knowledge spaces that facilitate equitable dialog between researchers and the public. In this way, we consider epistemic changes as a basis for broader transformations toward epistemic justice, including redistribution of power allocations and adaptations of organizational structures and procedures.

The issue remains how to introduce such changes in practice. In her analysis of institutional changes, Boyce (59), p. 130 proposes three groups of activities to support long-lasting changes: *inquiry and dialog*; *action learning*—practical problem-solving activities; and *embedding changes in the structures, systems, and cultures of the institution*. These activities are consistent with our thoughts on the *third-order* changes in research institutions. While it is hard to find a documented large-scale example of such changes concerning both the research institution and PPIE process, we will outline directions and examples close to our understanding of third-order changes.

## 5 Proposed directions

Our first proposed direction is for an increased focus on PPIE within academic teaching (10, 65). It consists of two-dimensional education: about the concepts and theories of PPIE, and also by gaining living experience in learning-by-doing approach, for example, meetings with patients or supervised PPIE projects (65). It could be combined with efforts to raise ethical awareness among researchers (53, 54), as well as raising the competences of early-stage researchers (66). Integrating elements of PPIE into academic curricula may have a twofold effect on research institutions. On the one hand, it may enhance future professionals’ meta-awareness about various sets of epistemic resources within contemporary society, increasing contributory justice, as well as broaden theoretical knowledge and raise socio-cultural sensitivity. Furthermore, it may counter persistent false beliefs and unsupported claims based on existing prejudices, such as those about Black people having higher pain tolerance than white people (67) or Deaf people having more concrete minds than hearing people (68), conducing the mitigation of testimonial injustice. This change encompasses mainly first- and second-order changes, including modifications of existing teaching strategies and broadening the educational resources with new perspectives, respectively. It may contribute to future third-order changes at research institutions, if these professionals-to-be would reorient their future workplaces toward a more PPIE-oriented approach. On the other hand, the PPIE-oriented education may directly transform institutional procedures and structures. Formalized within curricula, it may serve as a demonstration of institutional commitment to PPIE, diversity, and accessibility, affecting the willingness and self-efficacy among staff (9). Thus, it may be a step toward grounding PPIE within the institutional ethos and subsequent third-order change. Thomas et al. (10) and Churcher (36) suggest also a reform of teaching conditions within academia, by reformulating teachers’ roles, learner–teacher relations, and inviting scholars with minority/lived experience background. Academic staff with diverse sociocultural background may support sustainability of institutional changes and promote academic careers among people from diverse backgrounds [e.g., scholars with lived experience (69); Deaf people at academia: (70)]. Moreover, these activities may contribute to mitigating mistrust of historically marginalized groups toward research in general and facilitate engagement processes (46). Therefore, efforts focused on academic teaching may create favorable conditions for structural changes in institutional epistemic resources, both by future generations and through formalized curricula. It is likely that professionals trained in participatory approaches would be more eager to establish knowledge spaces in their research projects and have greater awareness about the different forms of knowledge among social groups.

Further direction relates to the implementation of culturally appropriate and decolonized approaches to research. Examples of such approaches are the *Indigenous Terms of Reference*—the set of principles which should be followed when doing research with a specific community (71); *Deaf studies*—an interdisciplinary field related to Deaf community, sign languages, and deafness (72); or *Kaupapa Māori* research—research grounded in Māori values and customs, by Māori, for Māori and with Māori (73). *Kaupapa Māori* is a distinct example, as it is explicitly incorporated into New Zealand Aotearoa’s higher education, aiming also to increase share of Māori population in research enterprises (74). These approaches and movements interact with mainstream scientific paradigms and negotiate space for epistemological diversity in science. Their formalization occurs through several research teams and institutes dedicated to various underrepresented perspectives. The question remains, whether in practice they interact and coexist with mainstream paradigms or both sides function rather separately (75). However, they have already raised meta-awareness about the various sets of epistemic resources shared by, for example Māori and non-Māori people, also at the institutional level. Thus, their presence facilitates ensuring contributory justice in research and seems to fit into the scope of third-order changes at specific research institutions. Such activities allow for establishing knowledge spaces in research and ease acceptance of PPIE approaches. Another similar postulated practice is *epistemic apprenticeship,* coined by Churcher (36). It concerns reorienting members from marginalized groups as mentors for those who are currently in power. Churcher (36), p. 10 stresses the need to institutionalize this practice to “expose and disrupt the epistemic solipsism and arrogance of privileged subjects through challenging imaginaries that sustain unspoken and uncritical attachments to dominant ways of knowing and being.” We think, that for such enterprise to be successful, it should begin with recognizing existence of diverse sets of epistemic resources—for example, Māori and non-Māori or neurodivergent and neurotypical. Thus, it requires third-order changes at research institutions. For such changes to be sustainable, these practices should be embedded in institutional ethos, to enable research institutions to influence behaviors of their members. It would allow to treat epistemic resources of unprivileged group members with respect, not only as a supportive “tool” for mainstream ones, therefore contributing to the contributory justice.

These directions can be linked with the idea of scientific/intellectual movements (SIM) which are collective efforts to advance research projects despite the opposition from scientific community (43, 76). They may serve as a means for broadening epistemic horizons of scientific institutions through identifying existing biases and providing new perspectives (77). SIMs can transform general ideas or values (e.g., democratic science or empowerment of marginalized groups) through research projects into a form of evidence [e.g., community-based research on experiencing dementia in Deaf community; (51) or research analyzing scientists’ mental health at academia; (69)]. Such movements are by definition “bottom-up” and their impact on institutional epistemic resources may be temporary without the support of authorities and senior staff (23, 59). However, they are grounded in local experiences and knowledge. SIMs may become vehicles for highlighting local hot spots within specific institutions which should be addressed, for example, frequent instances of testimonial injustice which lead to silencing a specific social group and precluding broader recognition of its experiences. SIM, being examples of non-institutionalized movements, can function as a starting point for more robust, second- and third-order changes which affect whole institution. Let us consider a hypothetical example of a research project on Deaf people’s research experiences. The study consists of focus groups and questionnaires discussing research accessibility, Deaf people’s relations with researchers and attitudes toward healthcare research. Started by hearing researchers, research team is broadened to include Deaf community members. Outcomes of the project highlight existing injustice and imply panel discussions with the authorities of the healthcare faculty. This in turn starts accessibility initiatives for whole faculty staff, conducted by Deaf educators. Then, Deaf people are hired on research and administrative positions. A debate on inclusion of Deaf epistemology into faculty’s curricula begins—the range of teaching methods is broadened to accommodate Deaf people’s visual and spatial modes of cognition. The faculty starts to diversify teaching programs, introduce bilingual spoken-signed newsletters, and invite Deaf students to its courses. It also creates a number of mixed Deaf-hearing research teams which facilitate cooperations with local community. It results in a dialog between the recent evidence-based knowledge and contextualized experiences of local Deaf community. This simplified and idealistic vision combines bits from each proposed direction. It begins with minor first- and second-order changes and then gradually leads to a third-order change, which broadens institutional epistemic resources and increases staff awareness. Many of such practices are already introduced within Deaf studies faculties. They contribute to transforming institutional decision-making procedures and establishing research institutions as a knowledge space open to PPIE.

## 6 Implications and challenges

The proposed directions can serve as a practical basis for analyzing the implications, limitations, and conditions for success of the conceptual framework. We believe that it has a real-life applicability; however, empirical research is needed. Deployment of all three orders of change at research institutions is one of necessary conditions for sustaining knowledge spaces for PPIE. We gathered and contextualized within PPIE practice only some directions, which can be further developed and adapted into local contexts. An increased focus on institutional epistemic features and practical application of third-order changes would enhance our understanding of epistemic processes within institutions. Consequently, this could contribute to better theorization of research institutions and structural epistemic injustice, leading to the establishment of a learning loop.

We recognize that proposed framework may encounter certain challenges. We would like to address a few of them. First, an issue of tensions between transformative roots of PPIE, especially participatory action research, and conservative structure of scientific institutions. A bottom-up movement, grounded within the virtue of social justice can easily become *a means to an end* and lose its transformative goals within the institution, which is focused merely on the quality of outcomes (78, 79). We acknowledge such risks, but no approach or paradigm is free of the risk of maladaptation in practice or misinterpretation [e.g., unintended disproportions within the three components of evidence-based medicine; (41)]. Thus, efforts of all involved agents are needed to stay on the track and consider all rationales behind the process. An increased focus on the role and epistemic features of institutions is crucial to hamper such risks.

This moves us also to the issue of feasibility and practicability of such changes. Third-order changes require significant efforts. Epistemic resources, both individual and collective, are resilient to change (18); thus, it is likely to expect the will to retain *status quo*. Moreover, changes in epistemic resources are not a sufficient condition for sustaining knowledge spaces. Changing a research institution requires all its staff to make significant efforts—organizational, financial, and educational, to implement and sustain a third-order change (59). In addition, such changes affect power relations and implicit biases in research institutions, as well as between public members and researchers (9, 30, 36). This pertains not only to the creation of knowledge spaces for PPIE but also to the idea of PPIE in general. Researchers’ attitudes may be ambivalent, especially among senior staff (30, 80). In addition, some funders and policymakers remain reluctant toward increasing active role of the members of public in research projects (32). While PPIE is increasingly acknowledged, several questions and uncertainties remain regarding its implementation (2). These are strong arguments restricting our proposition. While we argue for changes, we are also aware that the third-order change for knowledge spaces in PPIE may be an ideal, possible to reach to a varying degree. In addition, in complex institutions, such as universities, changes may be achieved within years or decades (59). They may start with first- or second-order changes and gradually reach more endorsement. Regarding PPIE, the process has already begun.

Another issue concerns downplaying the role of individual researchers and research teams, as well as hindering bottom-up initiatives. Institutionalization may easily become formalization, thus increasing formal barriers to researchers who conduct PPIE research. However, we argue that the features of third-order changes would allow for countering such issues. We acknowledge the role of individual researchers for PPIE, as well as sometimes conflicting values between participatory research and academia (79, 81). Researchers are crucial part of this process—they realize the knowledge spaces and represent academia in often very difficult contexts. Thus, changes within institutions should be holistic, involving the staff from each level of institutional hierarchy. They should integrate bottom-up and top-down movements, with the support of deliberate consideration of institutional epistemic features. We also emphasize that we do not want to promote a unified, monolithic approach to PPIE at research institutions. We recognize that each research institution has specific features and operates in a specific context, to which it should respond with its best possible understanding (14, 56), but, without further deliberation on general (epistemic) features of academic structure, research institutions may support PPIE in an (epistemically) harmful and vicious way (64, 79). This support, as well as the introduction of third-order changes, should be fitted to local contexts to facilitate the process and mitigate the risk of epistemic harms.

## 7 Conclusion

Within the study, we aimed to provide a conceptual framework for analyzing sociocultural context of institutional changes supporting establishing knowledge spaces and introducing PPIE practice. Ethical research conduct, especially in the PPIE context, requires considering various forms of knowledge present in contemporary society and examine existing biases. We argue that deep third-order changes, which aim to establish institutional meta-awareness about different forms of knowledge, are necessary (but not sufficient) to facilitate ethical PPIE practice. Research institutions—as active collective agents in the process—should make efforts to sustain favorable conditions for PPIE and mitigate possible harms to involved public members.

There is a need for empirical research which will operationalize and validate our proposal. This could take the form of case studies of specific changes within research institutions. In addition, the focus on institutional features is necessary to strengthen the theoretical basis for conceptualizing and planning changes. We need interdisciplinary collaboration which would enable the analysis of epistemic, social, political, economic, and organizational aspects of institutions. Furthermore, evaluative research is necessary to assess implementation of changes in practice.

Together with ethical inclusion and accessible research processes, the focus on research institutions is crucial to improve the PPIE conduct. While deep institutional changes aiming at establishing knowledge spaces may be a long-term ideal, they seem essential for fostering epistemically just and impactful research practice.

## Data Availability

The original contributions presented in the study are included in the article/supplementary material, further inquiries can be directed to the corresponding author.

## Footnotes

^1^Harrington et al. (1) use the term “patient engagement.” However, it matches our understanding of PPIE. For discussion of the differences in terminology, please see (1, 3).

^2^These groups are not mutually exclusive. For example, there may be patient-activists who conduct research or researchers who are also patients. However, being positioned as a layperson in the opaque context of (health) research institution can nevertheless be vulnerabilising, existing power differences make such distinction useful; see (31, 82).
